# Trajectories of spherical equivalent refraction from grades 1 to 4 in Chinese children

**DOI:** 10.1186/s12889-023-17420-x

**Published:** 2023-12-13

**Authors:** Yanzhi Li, Lan Guo, Jiayu Zhang, Xianghua Tang, Feng Zhao, Yin Hu, Yangfeng Guo, Xueying Du, Xiao Yang, Ciyong Lu

**Affiliations:** 1 Department of Medical Statistics and Epidemiology, School of Public Health, Sun Yat-Sen University, 74 Zhongshan Rd 2, Guangzhou, 510080 China; 2 State Key Laboratory of Ophthalmology, Zhongshan Ophthalmic Center, Sun Yat-Sen University, 54 Xianlie South Road, Guangzhou, 510060 China; 3 Health Promotion Center for Primary and Secondary Schools of Guangzhou Municipality, Guangzhou, China

**Keywords:** Myopia, Spherical equivalent refraction, Trajectories, Public primary school children, China

## Abstract

**Background:**

The development trajectories of children’s SER remain unknown. This study aimed to characterize spherical equivalent refraction (SER) trajectories during grades 1–4 in Chinese children.

**Methods:**

This prospective cohort study included 1226 first-grade non-myopic children from 12 public primary schools, randomly selected in two districts in Guangzhou, China. From November 2018 to March 2022, four-wave ocular examinations and questionnaire surveys have been completed. The group-based trajectory modeling was used to explore SER trajectories in grades 1–4.

**Results:**

All five trajectories showed an upward trend and rose faster after grade 2. Children in the sharp-developing (*n* = 44), high-developing (*n* = 136), and rapid-developing (*n* = 237) myopia groups developed myopia before grades 2, 3, and 4, respectively. Their SER development speed remained at a relatively high level after myopia, almost consistent with that before myopia. Children in the moderate-developing (*n* = 418) and low-developing (*n* = 391) non-myopia groups did not develop myopia before grade 4. Some characteristics in grade 1 were independently associated with SER trajectories, including sex, axial length, number of parents with myopia, residence, academic achievement, and the duration of outdoor activity. Based on the baseline characteristics, we established the model predicting the probability of children belonging to each group.

**Conclusions:**

Myopia interventions are best started in grade 1 or preschool age. If interventions are not taken in time, the latest intervention window might be in grades 1, 2, and 3 for children with a high probability of belonging to the sharp-developing, high-developing, and rapid-developing myopia groups, respectively. The above probabilities might be predicted using the model we established. Moreover, the interventions for myopic children shouldn’t be ignored.

**Supplementary Information:**

The online version contains supplementary material available at 10.1186/s12889-023-17420-x.

## Introduction

Myopia has become a serious public health issue, especially in some East and Southeast Asian countries [1]. It is estimated that by 2050, 50% of the global population will be myopic [2]. A significant concern of myopia is its progression to high myopia [1]. High myopia can lead to some sight-threatening complications including glaucoma, retinal detachment, and macular hole [3]. As the prevalence of myopia increases, the age of onset is earlier [2], especially in China [4]. Since myopia typically stabilizes in mid-to-late adolescence [5], myopic children with earlier onset tend to experience a longer duration to develop high myopia [6]. A retrospective study suggests that all 10-year-old (approximately grade 4) children with spherical equivalent refraction (SER) ≤ -3.0 diopter (D) develop high myopia (i.e., SER ≤ -6.0 D) in adulthood [7]. Thus, it is of importance to prevent the onset and progression of myopia in children.

In recent years, some interventions for myopia have been emerging [8, 9], but their effects are mixed [10], which may be caused by implementation time points or children in different grade levels. Moreover, the resources are limited and the implementation of interventions is also difficult to some extent. Therefore, two key steps to reduce the global burden of myopia are to identify high-risk children and appropriate intervention windows [11, 12]. Myopia can appear as early as primary school [12]. An in-depth understanding of SER trajectories during primary school may help identify children developing myopia at different times, which can provide clues for accomplishing the aforementioned goals. Since the grade level, rather than age, is a risk factor for myopia progress [13], previous cross-sectional and longitudinal studies used changes in the sample means to characterize a single development trajectory of SER with grade levels [11–14]. However, because the onset, progress, and stability of myopia vary widely among children [10], the sample means cannot accurately reflect the actual developmental trajectories of SER. This limitation results in existing evidence not providing sufficient scientific basis for determining high-risk children and appropriate intervention windows.

The group-based trajectory modeling (GBTM), a specialized form of finite mixture modeling, may help overcome the current limitations. The approach considers discrepancies in development trajectories of the population and can identify individuals following similar development trajectories, as well as the number and pattern of trajectories [15]. The GBTM has a good fitting effect and has been widely used to explore trajectories of children’s physical examination indicators (e.g., body mass index and blood pressure) [16, 17]. Additionally, based on baseline characteristics, the GBTM also performs well in predicting the probability that an individual belongs to each group [15]. Thus, by using the GBTM, we will more accurately describe the actual development trajectories of SER (e.g., low-developing, high-developing, and sharp-developing) and can predict the probability of children belonging to each group. Our findings may provide new insights for identifying high-risk children and appropriate intervention windows.

In this study, we hypothesized that children’s SER exhibited diverse development trajectories. Using longitudinal data of SER after cycloplegia, this study aimed to (1) identify the number and pattern of SER trajectories from grade 1 to grade 4 in Chinese children; (2) determine baseline characteristics associated with SER trajectories; and (3) establish a model predicting the probability of children belonging to each trajectory.

## Materials and methods

### Study design and participants

This ongoing cohort study was initiated from November to December 2018 to investigate the annual incidence, progression, and risk factors of myopia among Chinese schoolchildren. Six public primary schools were randomly selected from Panyu District (including 36 public primary schools) and Huadu District (including 38 public primary schools) in Guangzhou, China, respectively. Figure S1 shows the location of 12 public primary schools. According to the 2018 census data, Panyu District and Huadu District ranked 4th and 8th in the socio-economic ranking of Guangzhou’s 11 districts, respectively. All first-grade children in selected schools were invited to participate in this study except for children with strabismus, amblyopia, history of ocular surgery, other ocular conditions, or serious systemic diseases. A total of 1525 first-grade children participated in this study, with a response rate of 85.7%. These children were followed up annually in November and December, but the third follow-up was from December 2021 to March 2022, which was postponed due to COVID-19. To date, three follow-up visits have been completed. This study was approved by the Sun Yat-sen University, School of Public Health institutional review board and conducted in accordance with the Declaration of Helsinki. All children gave written informed consent signed by their parents or guardians.

To explore SER development of non-myopic children, we excluded 85 myopic (SER ≤ -0.5 D) children at baseline. Generally, the trajectory analysis requires at least three measurements of SER within each child [15], so 114 children with less than three measurements of SER were excluded. Ultimately, this study included 1226 children, 841 of whom participated in four SER examinations and 385 of whom participated in three SER examinations (Fig. S2).

### Ocular examinations

During the school day, a group of trained optometrists and ophthalmologists conducted ocular examinations using standardized protocols in 12 schools. The research team carried out the cover-uncover tests at both near and distance to identify strabismus and used slit lamp and direct ophthalmoscopy to detect abnormalities in the anterior and posterior segments. The axial length was measured using non-contact partial-coherence laser interferometry (IOLMaster 500; Zeiss). Cycloplegic autorefraction was conducted using a desktop autorefractor (KR8800; Topcon Corp). Cycloplegia was induced using 3 drops of 1% cyclopentolate with an interval of 5 min. Refractive measurement was performed after sufficient cycloplegia (absence of light reflex and a dilated pupil at least 6 mm in diameter). For all examinations, each eye was measured three times and the mean for each eye was calculated. During the baseline and follow-up surveys, the same team used the same equipment to perform the ocular examinations. SER was calculated as the sum of spherical power and half of the cylinder.

### Questionnaire investigations

The e-questionnaire was sent to the parents or guardians within one week after ocular examinations. Based on previous studies [3, 11, 12, 18–22], variables potentially associated with myopia were evaluated. Sociodemographic factors included sex, age, residence, family monthly income, and academic performance. Lifestyle factors included reading distance, distance to television, the duration of near vision, the duration of outdoor activity, the intake frequency of sugary drinks, and sleep quality. The duration of near vision was measured by the question: “On average, how much time does your child spend doing homework, reading extracurricular books, watching TV, using the computer, playing board games, and playing musical instruments per day?”. The duration of outdoor activity was assessed by the question: “On average, how much time does your child spend on outdoor activities (including cycling, going to the park, walking around, and doing sports) per day?”. The daily duration for near vision and outdoor activity was calculated using the following formula: (weekday time × 5 + weekend time × 2) / 7. Sleep quality was assessed using the 33-item Children’s Sleep Habits Questionnaire. The total score ranges from 33 to 99, with higher scores indicating poorer sleep quality [23]. The Children’s Sleep Habits Questionnaire has been widely used to measure the sleep quality of Chinese children, with a Cronbach coefficient of 0.75 [24]. Other factors included the number of parents with myopia, the mother’s childbearing age, the mode of delivery, and the duration of breastfeeding.

### Statistical analyses

All statistical analyses were performed using Stata version 17.0 (Stata Corporation, College Station, TX, USA). Because pairwise Pearson correlation showed a high correlation between SER of two eyes (*r* = 0.9; *P*-value < 0.001), data from the right eye was used in all analyses. The GBTM with a censored normal distribution was used to explore SER trajectories with grade levels. The Stata plugin named “traj” was utilized to perform the GBTM [25]. This study conducted a total of four surveys (i.e., one baseline survey and three follow-up surveys). From a mathematical perspective, four points can determine at most one cubic function, so trajectories may be cubic, quadratic, or linear functions. When the number of trajectories is the same, the cubic function (i.e., SER = *β*_0_ + *β*_1_ × grade + *β*_2_ × grade^2^ + *β*_3_ × grade^3^) usually has the best fit, followed by the quadratic function (i.e., SER = *β*_0_ + *β*_1_ × grade + *β*_2_ × grade^2^) and the linear function (i.e., SER = *β*_0_ + *β*_1_ × grade). The actual situation of the data was also like this (Tables S1, S2 and S3). Referring to Nagin’s suggestion [15], the selection steps and criteria of the optimal model are as follows. The first step was to use the cubic function (i.e., SER = *β*_0_ + *β*_1_ × grade + *β*_2_ × grade^2^ + *β*_3_ × grade^3^) to identify the optimal trajectory number by fitting 1–8 groups in turn. The optimal number was determined according to the following criteria: (1) 2*Δ Bayesian information criterion (BIC) > 10; (2) average posterior probability of assignment (AvePP) for each group > 0.7; (3) odds of correct classification (OCC) for each group > 5; and (4) percentage of children for each group > 2% [26, 27]. The second step was to identify the optimal polynomial degree for each group by reducing the polynomial order until the highest order polynomial coefficient was statistically significant (*P*-value < 0.05) [28]. Widely used for model selection in general, the BIC is the recommended criterion to determine the number of trajectory groups. To compare the BIC from two competing models, the Bayes factor can be used. For example, there is the Bayes factor comparing model *i* + *1* and model *i*. Model *i* + *1* is an *i* + *1*-group model and model *i* is an *i*-group model. The Bayes factor measures the odds of each of the two competing models being the correct model. It is computed as the ratio of the probability of model *i* + *1* being the correct model to the probability of model *i* being the correct model. A Bayes factor of 1 implies that the models are equally likely, whereas a Bayes factor of 10 implies that model *i* + *1* is 10 times more likely than model *i*. Computation of the Bayes factor is in general very difficult and indeed commonly impossible [29]. Alternatively, the simpler BIC log Bayes factor approximation, 2*log(Bayes factor _*i*+*1*_) ≈ 2*ΔBIC, may be applied, where ΔBIC is the difference between the BIC_*i*+*1*_ for the more complex model *i* + *1* and the BIC_*i*_ for the simpler model *i*. For this approximation, the value of 2*ΔBIC greater than 10 provides very strong evidence against the simpler model (i.e., model *i*). AvePP is the group-specific average posterior probability. For example, there are a total of five trajectories. The GBTM estimates the probability that each individual belongs to five trajectories. The individual belongs to the group with the highest probability. The average probability of individuals in group *j* belonging to the group *j* is AvePP of group *j*. OCC is the odds of correct classification for group *j*. The calculation formula for OCC is as follows: ${\mathrm{OCC}}_j=\frac{{\mathrm{AvePP}}_j/\left(1-{\mathrm{AvePP}}_j\right)}{{\widehat\pi}_j/\left(1-{\widehat\pi}_j\right)}$. ${\widehat\pi}_j$ is the estimated probability of group *j*. Nagin has found that AvePP of at least 0.7 for all groups and OCC of at least 5 for all groups are indicative of a model having high assignment accuracy [15].

Weighted multinomial logistic regression was used to estimate the crude association of baseline characteristics with SER trajectories. These regressions were weighted by the probability of group membership to account for measurement error introduced by the probabilistic nature of group assignment [27]. The group with the least SER progress was selected as the reference group. Baseline characteristics with a crude statistical significance (*P*-value < 0.10) correlation with SER trajectories were included in the weighted multivariable logistic regression. The forward-stepwise regression (threshold *P*-value < 0.20) was then used to select uniquely baseline characteristics, keeping age and sex [30]. In addition, the above-selected baseline characteristics were included within “traj” framework as baseline risk factors to establish a model calculating the probability of membership in each group.

## Results

There were no significant differences in the distribution of sex, age, residence, family monthly income, and academic achievement between all first-grade non-myopic children and those included in the analysis (Table S4). Among 1226 children, 660 were boys, 850 were 6 years old, 793 were from urban regions, 760 were from high-income families, and 659 had excellent academic performance.

**Fig. 1:**
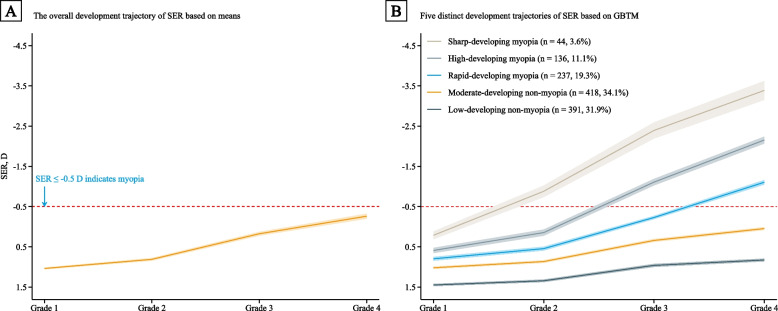
SER trajectories from grades 1 to 4 in Chinese children. The trajectories are shown in solid lines, and the 95% confidence intervals are shown in shadow. Abbreviations: SER, spherical equivalent refraction; D, diopter; GBTM, Group-based trajectory modeling

**Fig. 2:**
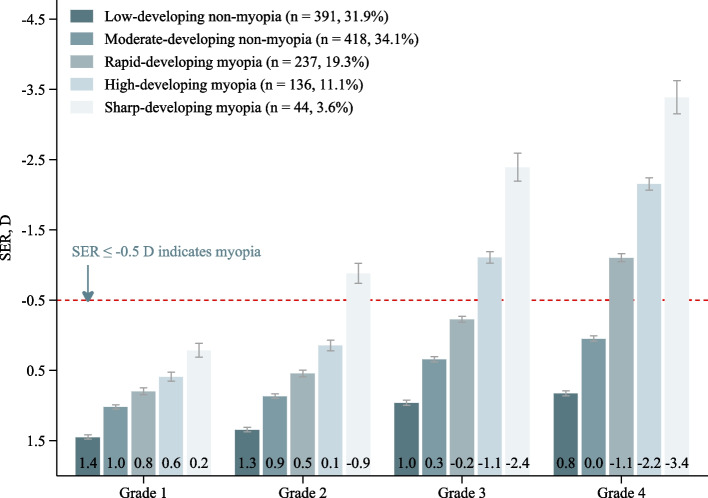
The actual SER mean and 95% CI for children in each trajectory during grades 1 − 4. Abbreviations: SER, spherical equivalent refraction; D, diopter; CI, confidence interval

The overall trajectory of SER based on the sample means showed an upward trend, rose at a faster rate after grade 2, and did not reach the threshold (i.e., SER ≤ -0.5 D) of myopia in grade 4 (Fig. 1A). By using a cubic function (i.e., SER = *β*_0_ + *β*_1_ × grade + *β*_2_ × grade^2^ + *β*_3_ × grade^3^) to fit 1–8 groups in turn, we found that the optimal number of trajectories was five (Table S1). The coefficients of the cubic terms of the five trajectories were statistically significant (all *P*-values < 0.001). Thus, according to the above model selection criteria, the optimal number was five groups and all groups’ optimal polynomial degree was cubic (i.e., SER = *β*_0_ + *β*_1_ × grade + *β*_2_ × grade^2^ + *β*_3_ × grade^3^). All SER trajectories showed an upward trend and rose faster after grade 2 (Figs. 1B and 2). Three trajectories developed myopia in different grades and were all labeled as myopia trajectories (34.0% of the sample). The sharp-developing group started at a mean SER of 0.2 D in grade 1 and developed myopia before grade 2. The high-developing group started at a mean SER of 0.6 D in grade 1 and developed myopia before grade 3. The rapid-developing group started at a mean SER of 0.8 D in grade 1 and developed myopia before grade 4. The SER of children in three myopic trajectories remained at a relatively high level after myopia, which was almost the same as that before myopia. The other two trajectories did not develop myopia before grade 4 and both were labeled as non-myopia trajectories (66.0% of the sample). The moderate-developing group was characterized by a moderate SER growth rate, from a mean SER of 1.0 D in grade 1 to a mean SER of 0.0 D in grade 4. The low-developing trajectory group was characterized by a relatively stable SER, starting at a mean SER of 1.4 D in grade 1 and leveling off at 0.8 D in grade 4.

**Fig. 3:**
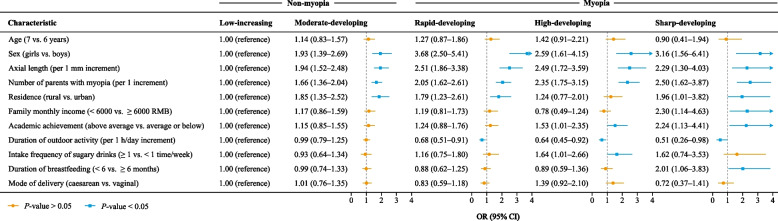
Final multivariable model predicting SER trajectories. Abbreviations: SER, spherical equivalent refraction; D, diopter; OR, Odds ratio; CI, confidence interval; RMB, renminbi (to convert to US dollars, multiply by 0.15)

Table 1 presents the baseline characteristics of children across SER trajectories. There were significant statistical differences in the distribution of axial length, sex, residence, family monthly income, academic achievement, and the number of parents with myopia (*P*-value < 0.05). The correlation between baseline characteristics was weak (Table S5). The results of univariate logistic regression are shown in Table S6, Ultimately, forward-stepwise logistic regression included age, sex, axial length, the number of myopic parents, residence, family monthly income, academic achievement, the duration of breastfeeding, the duration of outdoor activity, the intake frequency of sugary drinks, and the mode of delivery (Fig. 3). Specifically, referenced with the low-developing non-myopia group, girls, longer axial length, and greater number of myopic parents were risk factors for the other four groups. Greater duration of outdoor activity was associated with three myopia trajectories. Excellent academic achievement was a risk factor for the high-developing and sharp-developing myopia groups.

**Table 1:** Baseline characteristics of 1226 children according to five trajectories of spherical equivalent refraction^a^

**Characteristic**	**Total (*n* = 1226)**	**Non-myopia**		**Myopia**			***P*-value**^†^
		**Low-developing (*n* = 391)**	**Moderate-developing (*n* = 418)**	**Rapid-developing (*n* = 237)**	**High-developing (*n* = 136)**	**Sharp-developing (*n* = 44)**	
Spherical equivalent refraction, mean (SD), D	1.0 (0.5)	1.4 (0.3)	1.0 (0.3)	0.8 (0.4)	0.6 (0.4)	0.2 (0.3)	< 0.001
Axial length, mean (SD), mm	22.7 (0.7)	22.6 (0.6)	22.8 (0.6)	22.8 (0.7)	22.9 (0.8)	22.7 (0.6)	< 0.001
Sex							< 0.001
Boys	660 (53.8)	238 (60.9)	230 (55.0)	103 (43.5)	69 (50.7)	20 (45.5)	
Girls	566 (46.2)	153 (39.1)	188 (45.0)	134 (56.5)	67 (49.3)	24 (54.6)	
Age							0.530
6 years	850 (69.3)	281 (71.9)	289 (69.1)	160 (67.5)	88 (64.7)	32 (72.7)	
7 years	376 (30.7)	110 (28.1)	129 (30.9)	77 (32.5)	48 (35.3)	12 (27.3)	
Residence							0.009
Urban	793 (64.7)	270 (69.1)	253 (60.5)	145 (61.2)	100 (73.5)	25 (56.8)	
Rural	433 (35.3)	121 (31.0)	165 (39.5)	92 (38.8)	36 (26.5)	19 (43.2)	
Family monthly income							0.026
< 6000 RMB	466 (38.0)	150 (38.4)	167 (40.0)	91 (38.4)	36 (26.5)	22 (50.0)	
≥ 6000 RMB	760 (62.0)	241 (61.6)	251 (60.1)	146 (61.6)	100 (73.5)	22 (50.0)	
Academic achievement							0.012
Average or below	659 (53.8)	232 (59.3)	227 (54.3)	121 (51.1)	61 (44.9)	18 (40.9)	
Above average	567 (46.3)	159 (40.7)	191 (45.7)	116 (49)	75 (55.2)	26 (59.1)	
Reading distance							0.643
< 20 cm	259 (21.1)	84 (21.5)	92 (22.0)	40 (16.9)	32 (23.5)	11 (25.0)	
20–24 cm	530 (43.2)	166 (42.5)	186 (44.5)	102 (43.0)	58 (42.7)	18 (40.9)	
25–29 cm	299 (24.4)	97 (24.8)	90 (21.5)	71 (30.0)	29 (21.3)	12 (27.3)	
≥ 30 cm	138 (11.3)	44 (11.3)	50 (12.0)	24 (10.1)	17 (12.5)	3 (6.8)	
Distance to television							0.667
< 1.0 m	65 (5.3)	24 (6.1)	17 (4.1)	13 (5.5)	7 (5.2)	4 (9.1)	
1.0–1.9 m	366 (29.9)	120 (30.7)	118 (28.2)	72 (30.4)	44 (32.4)	12 (27.3)	
2.0–3.0 m	565 (46.1)	186 (47.6)	199 (47.6)	102 (43.0)	61 (44.9)	17 (38.6)	
> 3.0 m	230 (18.8)	61 (15.6)	84 (20.1)	50 (21.1)	24 (17.7)	11 (25.0)	
Duration of near vision, mean (SD), min/day	196.4 (101.3)	202.4 (106.7)	191.3 (94.3)	195.5 (103.1)	197.1 (102)	192.7 (105.3)	0.643
Duration of outdoor activity, mean (SD), min/day	49.7 (37.2)	52.8 (40.7)	52.2 (38.7)	44.4 (31.7)	45.3 (30.4)	39.9 (31.1)	0.006
Intake frequency of sugary drinks							0.274
< 1 time/week	986 (80.4)	316 (80.8)	346 (82.8)	189 (79.8)	101 (74.3)	34 (77.3)	
≥ 1 time/week	240 (19.6)	75 (19.2)	72 (17.2)	48 (20.3)	35 (25.7)	10 (22.7)	
Sleep quality scores, mean (SD)	46.7 (9.1)	46.2 (8.3)	46.5 (8.5)	47.7 (9.9)	47.2 (10.8)	47.0 (12.0)	0.297
Number of parents with myopia							< 0.001
0	565 (46.1)	226 (57.8)	181 (43.3)	97 (40.9)	45 (33.1)	16 (36.4)	
1	430 (35.1)	123 (31.5)	166 (39.7)	79 (33.3)	47 (34.6)	15 (34.1)	
2	231 (18.8)	42 (10.7)	71 (17.0)	61 (25.7)	44 (32.4)	13 (29.6)	
Mother’s childbearing age							0.703
< 35 years	1138 (92.8)	361 (92.3)	387 (92.6)	225 (94.9)	125 (91.9)	40 (90.9)	
≥ 35 years	88 (7.2)	30 (7.7)	31 (7.4)	12 (5.1)	11 (8.1)	4 (9.1)	
Mode of delivery							0.063
Vaginal	651 (53.1)	205 (52.4)	222 (53.1)	138 (58.2)	59 (43.4)	27 (61.4)	
Caesarean	575 (46.9)	186 (47.6)	196 (46.9)	99 (41.8)	77 (56.6)	17 (38.6)	
Duration of breastfeeding							0.190
< 6 months	522 (42.6)	164 (41.9)	180 (43.1)	93 (39.2)	59 (43.4)	26 (59.1)	
≥ 6 months	704 (57.4)	227 (58.1)	238 (56.9)	144 (60.8)	77 (56.6)	18 (40.9)	

*Abbreviation*: *D* diopter, *SD* standard deviation, *RMB* renminbi (to convert to US dollars, multiply by 0.15)

^†^*P*-value was based on Pearson chi-squared tests or one-analysis of variance where appropriate

^a^Unless indicated otherwise, data are given as No. (%)

According to the article by Nagin [29], using the values for a child’s baseline characteristics and the fitted parameters (i.e., *β* values in Table 2), can calculate the probability of a child belonging to each group. The calculation process is described in Table 2. For example, a 7-year-old girl with an axial length of 22.5 mm and non-myopia parents, comes from a low-income family (i.e., family monthly income < 6000 RMB) in the rural region, has excellent academic achievement, was breastfed for less than 6 months, was born in a vaginal mode, takes part in outdoor activity for an hour every day, and drinks sugary drinks more than once a week. *H*_moderate-developing non-myopia_ = exp (1 × 0.24082 + 1 × 0.81305 + 22.5 × 0.84747 + 0 × 0.66239 + 1 × 0.81354 + 1 × 0.16655 + 1 × 0.25422 + 1 × 0.03839 + 0 × -0.08992 + 1 × -0.03378 + 0 × 0.02632 - 23.70648) = 0.09579. Similarly, *H*_rapid-developing myopia_ = 0.02362; *H*_high-developing myopia_ = 0.00175; and *H*_sharp-developing myopia_ = 0.01076. *P*_moderate-developing non-myopia_ = 0.09579 / (0.09579 + 0.02362 + 0.00175 + 0.01076 + 1) × 100% = 8.46%. Similarly, *P*_rapid-developing myopia_ = 2.09%; *P*_high-developing myopia_ = 0.15%; and *P*_sharp-developing myopia =_ 0.95%. *P*_low-developing non-myopia group_ = 1 - 8.46% - 2.09% - 0.15% - 0.95% = 88.35%. Thus, the probabilities that the girl belongs to the low-developing non-myopia group, moderate-developing non-myopia group, rapid-developing myopia group, high-developing myopia group, and sharp-developing myopia group are 88.35%, 8.46%, 2.09%, 0.15%, and 0.95%, respectively. The identifiable 7-year-old girl is just an example, not an actual child, which is for the readers to better understand the prediction process. There is no such child in our dataset. Any resemblance to a real person living or deceased will be a coincidence.

**Table 2:** Final model parameters and method to calculate the probability of membership in each of the 5 groups using baseline characteristics^a^

Characteristic	Parameter value (*β*)				
	**Non-myopia**		**Myopia**		
	**Low-increasing**	**Moderate-increasing**	**Rapid-increasing**	**High-increasing**	**Sharp-increasing**
Age (7 vs. 6 years)	Reference	0.24082	0.32208	0.46648	-0.13172
Sex (girls vs. boys)	Reference	0.81305	1.42135	1.10385	1.22421
Axial length (per 1 mm increment)	Reference	0.84747	1.04089	1.08898	1.03163
Number of parents with myopia (per 1 increment)	Reference	0.66239	0.86650	0.99335	0.96744
Residence (rural vs. urban)	Reference	0.81354	0.66664	0.38639	0.78424
Family monthly income (< 6000 vs. ≥ 6000 RMB)	Reference	0.16655	0.17961	-0.34635	0.82949
Academic achievement (above average vs. average or below)	Reference	0.25422	0.25955	0.55817	0.98654
Duration of outdoor activity (per 1 h/day increment)	Reference	0.03839	-0.38728	-0.42679	-0.63260
Intake frequency of sugary drinks (≥ 1 vs. < 1 time/week)	Reference	-0.08992	0.07105	0.52550	0.45167
Duration of breastfeeding (< 6 vs. ≥ 6 months)	Reference	-0.03378	-0.13313	-0.15072	0.72493
Mode of delivery (caesarean vs. vaginal)	Reference	0.02632	-0.16382	0.34495	-0.27137
Constant	Reference	-23.70648	-29.49446	-32.44119	-31.52807

*Abbreviations*: *RMB* renminbi (to convert to US dollars, multiply by 0.15)

^a^(A) for each column above, raise *e* to the power of [(axial length × value above) + … + (mode of delivery × value above) + constant]; (B) the estimated probability of membership in each group is equal to the number calculated for each column in step A divided by [1 + sum of all numbers (one for each column) calculated in step A]; and (C) the probability of being in the low-increasing non-myopia group (reference) is equal to 1 minus the sum of probabilities of being in the 4 other groups (from step B). Taken from equation 3 in the article by Nagin [29]

## Discussion

Using the longitudinal data of refraction with cycloplegia, this study found five distinct trajectories of SER during grades 1–4 among first-grade non-myopia children in China: two non-myopia trajectories (i.e., low-increasing and moderate-increasing) and three myopia trajectories (i.e., rapid-increasing, high-increasing, and sharp-increasing). Some baseline characteristics may be associated with SER trajectories, including gender, axial length, number of parents with myopia, residence, family monthly income, academic achievement, the duration for outdoor activity, the intake frequency of sugary drinks, and the duration of breastfeeding. Moreover, we established a model that can predict the probability of children belonging to each trajectory. These findings provide the scientific basis for identifying the individuals and appropriate timing of intervention to prevent myopia.

Before using GMTM, this study used the sample means to describe the overall development trajectory of SER. Consistent with the results of a longitudinal study from Anyang, China [12], SER showed an upward trend, rising faster after grade 2, and did not develop myopia in grade 4. However, another longitudinal study from Guangzhou, China found that SER developed more slowly after grade 2 and developed myopia before grade 4 [11]. Since the study from Guangzhou did not perform cycloplegic refraction, the assessment of SER was inaccurate, which may be the reason for the discrepancy [31]. Another more important reason is that it may be inappropriate to use the sample means to describe SER trajectories, since the onset, progression, and stability of myopia tend to vary considerably among children (i.e., heterogeneity) [10]. In contrast, this study used the GBTM to capture the heterogeneity of SER development [29], thus we more accurately characterized SER trajectories. On the whole, all five SER trajectories showed an upward trend and rose faster after grade 2. Taken together, the consistently rising trend of SER and the relatively high development speed of SER during grades 2–3 suggest that interventions should be started in grade 1 or preschool age.

Besides, the GMTM showed that distinct trajectories progressed at different rates and developed myopia at different grade levels, which provides new insights for determining high-risk children and appropriate intervention windows, and indicates that it may be inappropriate to utilize the sample means to describe SER trajectories. Specifically, 3.6% of children developed myopia before grade 2, and their SER was lower than -3.0 D in grade 4; 11.1% of children developed myopia before grade 3, with SER ranging from -1.5 D to -3.0 D in grade 4; and 19.3% of children developed myopia before grade 2, with SER of -0.5 D to -1.5 D in grade 4. The incidence in each grade is similar to that in another study from China [12], further supporting our findings. Three myopia trajectories indicate that the latest intervention windows should be in grades 1, 2, and 3 for children belonging to the above three groups, respectively. Without interventions before the aforementioned time, most of the above children may develop myopia. Another noteworthy event is that although 34.1% of children, the greatest percent, did not have myopia before grade 4, most of them may develop myopia in the future according to the current growth rate. Such children should also be taken seriously. Future studies with longer follow-ups time are needed to clarify their SER development trajectory in subsequent grades.

Furthermore, the SER of children in three myopic trajectories remained at a relatively high level after myopia, almost consistent with that before myopia. This phenomenon may be caused by insufficient attention to myopia children. One longitudinal study clarified the risk of 10-year-old (approximately grade 4) children with different SERs (i.e., ≥ -1.5 D to ≤ -0.5 D, > -3.0 D to < -1.5 D, and ≤ -3.0 D) developing high myopia (i.e., SER ≤ -6.0 D) in adulthood [7]. Combining the findings of the above study, children belonging to sharp-developing, high-developing, and rapid-developing myopia groups have 100%, 46.0%, and 32.6% risks of developing high myopia in adulthood, respectively. Thus, we should not ignore children who are already myopic and should intervene with them to slow down the progress of myopia. Otherwise, they have a high risk of developing high myopia in adulthood.

This study found that some baseline characteristics were related to the development of SER, which have been reported in previous studies [3, 11, 12, 18–22]. However, the factors affecting the occurrence of myopia in different grades are discrepant, suggesting that the inconsistencies in previous intervention studies are due to the fact that the children receiving the intervention in different grades [10]. Three genetically related factors (i.e., sex, axial length, and the number of myopic parents) were associated with all SER trajectories, indicating that genetic factors are crucial in the development of SER. Excellent academic performance was a risk factor for developing myopia before grades 2 (i.e. the sharp-developing group) and 3 (i.e. the high-developing group), but not before grade 4 (i.e. the rapid-developing group), and it had a greater impact on the development of myopia before grade 2 than that before grade 3, so we should pay more attention to SER development of children with excellent academic performance in the lower grades. It is noteworthy that the duration of outdoor activity, as a modifiable factor, was associated with three myopia trajectories. The underlying mechanism for outdoor activity to prevent myopia is that the high intensity, spectral composition, and wavelength of outdoor light may promote retinal dopamine release and vitamin D synthesis, thereby regulating SER growth [32]. To better prevent the occurrence and progress of myopia, increasing outdoor activity in schoolchildren should be advocated. More importantly, based on the characteristics, we established a model predicting the probability of children belonging to each group. This approach has been widely used to predict the future trajectory of a certain factor based on baseline characteristics [33, 34]. The model may provide valuable clues for determining the subjects of interventions at different times. At appropriate intervention windows, increasing the duration of outdoor activity, strengthening family health education, and consuming saffron might be effective intervention measures for myopia [8, 9, 35]. One strength is that based on the differences in children’s SER development, the GBTM was adopted to characterize five distinct SER development trajectories. The model selected had a good fitting effect, guaranteeing the reliability of the results. Another strength is that trained optometrists and ophthalmologists used the standardized scheme to measure children’s SER after cycloplegia, and the team and equipment were the same for each examination, which ensured the accuracy and comparability of SER. Nevertheless, several limitations should be noted. First, this study only described SER trajectories during grades 1–4. Future studies are warranted to explore SER trajectories throughout the school-age period. Second, because most baseline characteristics were self-reported, the potential reporting bias could not be eliminated. Third, we investigated students’ past behavioral habits, the potential recall bias should not be ignored. Fourth, the third follow-up was postponed due to COVID-19, so the SER trajectories we found may not be the normal development pattern. If the third follow-up date is the same as the first and second follow-up dates, the trajectories during grades 3–4 may be smoother than those we observed. Fifth, although we have explored the association between some variables and SER trajectories, some variables have not been considered, such as parents’ occupation and education level. Finally, our findings are limited to children in Guangzhou, China. Guangzhou is located in the south of China, which has a longer sunshine duration and ultraviolet intensity than the north of China, so this may limit the generalizability to the whole Chinese population. Moreover, it is necessary to confirm our findings among children in other countries.

In conclusion, this study suggests that there may exist five distinct SER trajectories during grades 1–4 in Chinese children. Effective interventions are best initiated in grade 1 or preschool age. If interventions are not taken in time, the latest intervention time may be in grades 1, 2, and 3 for children with a high probability of belonging to the sharp-developing, high-developing, and rapid-developing myopia groups, respectively. The above probabilities can be predicted using the model we established. Additionally, we should attach importance to children who are already myopic to prevent them from developing high myopia. Interventions of myopia may vary by grade level, but increasing outdoor activity should be advocated throughout school age. Our findings provide new insights for identifying high-risk children at different stages and appropriate intervention windows.

### Supplementary Information

**Additional file 1: Table S1.** The results of the group-based trajectory modeling fitting process: the cubic function. **Table S2.** The results of the group-based trajectory modeling fitting process: the quadratic function. **Table S3.** The results of the group-based trajectory modeling fitting process: the linear function. **Table S4.** Baseline characteristics of all children without myopia at baseline and children included in analysis. **Table S5.** The correlations for baseline characteristics of 1226 children. **Table S6.** Univariate model predicting five trajectories of spherical equivalent refraction. **Fig S1.** Map of schools’ locations in Guangzhou, China. **Fig S2.** Flowchart of children included in analysis.

## Data Availability

The datasets generated and analysed during the current study are not publicly available due to privacy constraints but are available from the corresponding author on reasonable request.

## Abbreviations

AvePP: Average posterior probability
BIC: Bayesian information criterion
CI: Confidence interval
D: Diopter
GBTM: Group-based trajectory modeling
OCC: Odds of correct classification
OR: Odds ratio
RMB: Renminbi (to convert to US dollars, multiply by 0.15)
SER: Spherical equivalent refraction
