# Supplementary material for: Trajectories of spherical equivalent refraction from grades 1 to 4 in Chinese children
Source: BMC Public Health. 2023 Dec 13;23:2500. doi: 10.1186/s12889-023-17420-x (PMC10720137; doi:10.1186/s12889-023-17420-x)
Supplement: Supplementary file 1 — Additional file 1: Table S1. The results of the group-based trajectory modeling fitting process: the cubic function. Table S2. The results of the group-based trajectory modeling fitting process: the quadratic function. Table S3. The results of the group-based trajectory modeling fitting process: the linear function. Table S4. Baseline characteristics of all children without myopia at baseline and children included in analysis. Table S5. The correlations for baseline characteristics of 1226 children. Table S6. Univariate model predicting five trajectories of spherical equivalent refraction. Fig S1. Map of schools’ locations in Guangzhou, China. Fig S2. Flowchart of children included in analysis. [file 12889_2023_17420_MOESM1_ESM.docx]

**Supplementary Materials**

**Title:** Trajectories of spherical equivalent refraction from grades 1 to 4 in Chinese children

**A running title:** Trajectories of spherical equivalent refraction

**Table S1.** The results of the group-based trajectory modeling fitting process: the cubic function
**Table S2.** The results of the group-based trajectory modeling fitting process: the quadratic function
**Table S3.** The results of the group-based trajectory modeling fitting process: the linear function

**Table S4.** Baseline characteristics of all children without myopia at baseline and children included in analysis

**Table S5.** The correlations for baseline characteristics of 1226 children.

**Table S6.** Univariate model predicting five trajectories of spherical equivalent refraction

**Fig S1.** Map of schools' locations in Guangzhou, China

**Fig S2.** Flowchart of children included in analysis

| **Table S1.** The results of the group-based trajectory modeling fitting process: the cubic function * | | | | | | |
| --- | --- | --- | --- | --- | --- | --- |
| **No. of groups** | **Polynomial degree** | **BIC** | **2*ΔBIC †** | **Proportion of participants (%)** | **AvePP (%)** | **OCC** |
| 1 | Cubic | -5590 |  | 100.0 | 100.0 |  |
| 2 | Cubic | -4365 | 2450 | 72.3 / 27.7 | 98.0 / 94.2 | 19 / 42 |
| 3 | Cubic | -3866 | 998 | 60.0 / 32.4 / 7.7 | 96.9 / 93.0 / 95.5 | 21 / 28 / 250 |
| 4 | Cubic | -3607 | 518 | 41.3 / 34.5 / 18.7 / 5.5 | 93.4 / 87.5 / 94.5 / 96.8 | 20 / 14 / 72 / 520 |
| **5** | **Cubic** | **-3511** | **192** | **31.9 / 34.1 / 19.3 / 11.1 / 3.6** | **89.9 / 86.2 / 89.0 / 89.3 / 97.5** | **19 / 12 / 32 / 70 / 1013** |
| 6 | Cubic | -3439 | 144 | 11.3 / 41.1 / 24.6 / 15.9 / 5.9 / 1.2 | 86.8 / 88.3 / 87.0 / 92.2 / 91.7 / 94.7 | 46 / 11 / 21 / 61 / 179 / 1352 |
| 7 | Cubic | -3417 | 44 | 10.8 / 40.7 / 22.9 / 15.5 / 5.9 / 2.7 / 1.5 | 88.3 / 88.0 / 85.7 / 90.2 / 92.0 / 79.2 / 92 | 55 / 12 / 20 / 51 / 187 / 114 / 743 |
| 8 | Cubic | -3354 | 126 | 8.2 / 34.2 / 24.6 / 13.1 / 5.1 / 9.6 / 4.3 / 1.0 | 87.0 / 87.2 / 82.6 / 82.5 / 76.8 / 89.9 / 95.9 / 98.8 | 69 / 14 / 15 / 31 / 61 / 83 / 508 / 8134 |
| * The best-fitting model is highlighted in bold characters.  † Value is evidence against the null hypothesis.  Abbreviation: BIC, Bayesian information criterion; AvePP, average posterior probability; OCC, odds of correct classification. | | | | | | |

| **Table S2.** The results of the group-based trajectory modeling fitting process: the quadratic function | | | | | | |
| --- | --- | --- | --- | --- | --- | --- |
| **No. of groups** | **Polynomial degree** | **BIC** | **2*ΔBIC †** | **Proportion of participants (%)** | **AvePP (%)** | **OCC** |
| 1 | Quadratic | -5601 |  | 100.0 | 100.0 |  |
| 2 | Quadratic | -4391 | 1210 | 73.2 / 26.8 | 97.7 / 94.6 | 16 / 47 |
| 3 | Quadratic | -3903 | 488 | 59.4 / 33.1 / 7.5 | 97.1 / 91.7 / 96.5 | 23 / 23 / 327 |
| 4 | Quadratic | -3650 | 253 | 40.8 / 35.2 / 18.5 / 5.5 | 93.1 / 87.1 / 93.7 / 97.1 | 19 / 13 / 64 / 564 |
| **5** | Quadratic | -3570 | 80 | 29.5 / 10.6 / 35.1 / 20.8 / 4.0 | 87.9 / 88.7 / 85 / 87.1 / 94.3 | 18 / 67 / 10 / 25 / 397 |
| 6 | Quadratic | -3485 | 85 | 11.3 / 5.4 / 15.8 / 41.5 / 24.7 / 1.3 | 86.1 / 93.2 / 89.7 / 86.7 / 86.1 / 93.2 | 42 / 235 / 47 / 10 / 19 / 1057 |
| 7 | Quadratic | -3447 | 38 | 7.7 / 9.5 / 4.4 / 36.3 / 24.8 / 16.5 / 0.8 | 83.4 / 87.8 / 95.5 / 84.9 / 80.6 / 84.4 / 95.2 | 54 / 68 / 450 / 10 / 13 / 27 / 2477 |
| 8 | Quadratic | -3412 | 35 | 10.5 / 7.5 / 4.5 / 9.8 / 34.5 / 26.2 / 6.2 / 0.8 | 75.3 / 84.3 / 95.6 / 88.2 / 85.6 / 79.8 / 74.4 / 96.5 | 26 / 59 / 458 / 70 / 12 / 12 / 38 / 3396 |
| † Value is evidence against the null hypothesis.  Abbreviation: BIC, Bayesian information criterion; AvePP, average posterior probability; OCC, odds of correct classification. | | | | | | |

| **Table S3.** The results of the group-based trajectory modeling fitting process: the linear function | | | | | | |
| --- | --- | --- | --- | --- | --- | --- |
| **No. of groups** | **Polynomial degree** | **BIC** | **2*ΔBIC †** | **Proportion of participants (%)** | **AvePP (%)** | **OCC** |
| 1 | Linear | -5605 |  | 100.0 | 100.0 |  |
| 2 | Linear | -4420 | 1185 | 72 / 28 | 98.2 / 93.7 | 20 / 39 |
| 3 | Linear | -3950 | 470 | 60 / 32.4 / 7.7 | 96.8 / 92.9 / 95.1 | 20 / 27 / 231 |
| 4 | Linear | -3720 | 230 | 41 / 34.8 / 18.7 / 5.5 | 93.3 / 87 / 94.6 / 97.2 | 20 / 13 / 73 / 590 |
| **5** | Linear | -3641 | 79 | 12.3 / 42 / 24.3 / 16.3 / 5.1 | 86 / 86.3 / 86.5 / 91.1 / 97.8 | 38 / 9 / 20 / 54 / 800 |
| 6 | Linear | -3574 | 67 | 11.1 / 16.2 / 40.9 / 24.8 / 5.7 / 1.3 | 86.5 / 91.9 / 87.8 / 86.4 / 93.2 / 92.6 | 45 / 58 / 11 / 20 / 222 / 945 |
| 7 | Linear | -3541 | 33 | 14.8 / 9.3 / 37.4 / 22.9 / 10.3 / 4.2 / 1.1 | 82.6 / 85 / 87.7 / 81.6 / 87.5 / 96.1 / 93.9 | 27 / 52 / 12 / 15 / 64 / 544 / 1499 |
| 8 | Linear | -3522 | 19 | 8.1 / 33.9 / 25 / 13.1 / 4.8 / 9.8 / 4.2 / 1.1 | 85.3 / 86.7 / 81.1 / 81 / 75 / 89.1 / 96.4 / 95.8 | 61 / 13 / 13 / 29 / 55 / 75 / 571 / 2203 |
| † Value is evidence against the null hypothesis.  Abbreviation: BIC, Bayesian information criterion; AvePP, average posterior probability; OCC, odds of correct classification. | | | | | | |

| **Table S4.** Baseline characteristics of all children without myopia at baseline and children included in analysis * | | | |
| --- | --- | --- | --- |
| **Characteristic** | **All students**  **without myopia**  **(n = 1440)** | **Students included**  **in analysis**  **(n =** **1226)** | ***P*-value †** |
| Sex |  |  | 0.596 |
| Boys | 790 (54.9) | 660 (53.8) |  |
| Girls | 650 (45.1) | 566 (46.2) |  |
| Age |  |  | 0.712 |
| 6 years | 989 (68.7) | 850 (69.3) |  |
| 7 years | 451 (31.3) | 376 (30.7) |  |
| Residence |  |  | 0.584 |
| Urban | 946 (65.7) | 793 (64.7) |  |
| Rural | 494 (34.3) | 433 (35.3) |  |
| Family monthly income |  |  | 0.815 |
| < 6,000 RMB | 541 (37.6) | 466 (38.0) |  |
| ≥ 6,000 RMB | 899 (62.4) | 760 (62.0) |  |
| Academic achievement |  |  | 0.914 |
| Average or below | 771 (53.5) | 659 (53.8) |  |
| Above average | 669 (46.5) | 567 (46.3) |  |
| * Data are given as No. (%). † *P*-value was based on Pearson chi-squared tests.  Abbreviations: RMB, renminbi (to convert to US dollars, multiply by 0.15). | | | |

| **Table S5.** The correlations for baseline characteristics of 1226 children. | | | | | | | | | | | | | | | | | | | | | | | |
| --- | --- | --- | --- | --- | --- | --- | --- | --- | --- | --- | --- | --- | --- | --- | --- | --- | --- | --- | --- | --- | --- | --- | --- |
|  | **1** | **2** | **3** | | **4** | **5** | | **6** | **7** | **8** | | | **9** | **10** | | **11** | **12** | | **13** | **14** | | **15** | **16** |
| Axial length | 1 |  |  | |  |  | |  |  |  | | |  |  | |  |  | |  |  | |  |  |
| Sex | -0.442 | 1 |  | |  |  | |  |  |  | | |  |  | |  |  | |  |  | |  |  |
| Age | 0.095 | -0.014 | 1 | |  |  | |  |  |  | | |  |  | |  |  | |  |  | |  |  |
| Residence | -0.057 | -0.021 | 0.014 | | 1 |  | |  |  |  | | |  |  | |  |  | |  |  | |  |  |
| Family monthly income | 0.021 | 0.045 | -0.080 | | -0.216 | 1 | |  |  |  | | |  |  | |  |  | |  |  | |  |  |
| Academic achievement | 0.017 | 0.104 | 0.116 | | -0.050 | 0.106 | | 1 |  |  | | |  |  | |  |  | |  |  | |  |  |
| Reading distance | 0.015 | 0.024 | 0.004 | | -0.024 | 0.028 | | 0.099 | 1 |  | | |  |  | |  |  | |  |  | |  |  |
| Distance to television | 0.003 | 0.012 | -0.008 | | -0.035 | 0.100 | | 0.056 | 0.254 | 1 | | |  |  | |  |  | |  |  | |  |  |
| Duration of near vision | 0.048 | -0.054 | -0.020 | | -0.018 | -0.078 | | -0.119 | -0.009 | -0.003 | | | 1 |  | |  |  | |  |  | |  |  |
| Duration of outdoor activity | 0.017 | 0.009 | -0.008 | | -0.077 | 0.057 | | 0.040 | 0.144 | 0.154 | | | 0.095 | 1 | |  |  | |  |  | |  |  |
| Intake frequency of sugary drinks | 0.065 | -0.061 | 0.025 | | 0.033 | -0.017 | | -0.086 | -0.033 | -0.078 | | | 0.195 | 0.012 | | 1 |  | |  |  | |  |  |
| Sleep quality scores | 0.010 | 0.011 | 0.025 | | -0.027 | -0.047 | | -0.095 | -0.151 | -0.117 | | | 0.229 | -0.088 | | 0.157 | 1 | |  |  | |  |  |
| Number of parents with myopia | 0.045 | 0.015 | -0.079 | | -0.157 | 0.212 | | 0.063 | -0.040 | 0.106 | | | -0.021 | -0.005 | | -0.044 | 0.002 | | 1 |  | |  |  |
| Mother's childbearing age | -0.004 | -0.020 | 0.031 | | -0.058 | -0.059 | | 0.009 | -0.024 | 0.024 | | | -0.045 | 0.044 | | -0.021 | 0.041 | | -0.060 | 1 | |  |  |
| Mode of delivery | 0.034 | -0.017 | 0.036 | | -0.086 | 0.057 | | 0.013 | 0.046 | 0.073 | | | -0.032 | 0.056 | | 0.053 | -0.055 | | 0.018 | 0.113 | | 1 |  |
| Duration of breastfeeding | 0.039 | 0.004 | 0.027 | | -0.004 | -0.075 | | 0.024 | 0.021 | -0.018 | | | 0.041 | 0.075 | | 0.061 | -0.014 | | -0.058 | -0.008 | | -0.074 | 1 |
| **Table S6.** Univariate model predicting five trajectories of spherical equivalent refraction | | | | | | | | | | | | | | | | | | | | |  |  |  |
| **Characteristic** | | | | **Odds ratio (95% confidence interval)** | | | | | | |  |  | | | | | | | | |  |  |  |
|  |  |  |  | **Non-myopia** | | |  | | | |  | **Myopia** | | |  | | |  | | |  |  |  |
|  |  |  |  | **Low-increasing** | | | **Moderate-increasing** | | | |  | **Rapid-increasing** | | | **High-increasing** | | | **Sharp-increasing** | | |  |  |  |
| Axial length (per 1 mm increment) | | | | 1.00 (Reference) | | | 1.49 (1.21–1.83) ‡ | | | |  | 1.54 (1.19–1.99) ‡ | | | 1.85 (1.33–2.59) ‡ | | | 1.42 (0.88–2.28) | | |  |  |  |
| Sex (girls vs. boys) | | | | 1.00 (Reference) | | | 1.30 (0.98–1.73) * | | | |  | 2.11 (1.52–2.94) ‡ | | | 1.54 (1.03–2.29) † | | | 1.91 (1.02–3.58) † | | |  |  |  |
| Age (7 vs. 6 years) | | | | 1.00 (Reference) | | | 1.18 (0.87–1.60) | | | |  | 1.29 (0.91–1.84) | | | 1.45 (0.95–2.21) * | | | 0.96 (0.47–1.94) | | |  |  |  |
| Residence (urban vs. rural) | | | | 1.00 (Reference) | | | 1.54 (1.15–2.06) ‡ | | | |  | 1.01 (0.72–1.42) * | | | 0.57 (0.37–0.88) | | | 1.59 (0.85–2.97) * | | |  |  |  |
| Family monthly income (< 6000 vs. ≥ 6000 RMB) | | | | 1.00 (Reference) | | | 1.08 (0.81–1.43) | | | |  | 0.99 (0.71–1.38) | | | 1.75 (1.13–2.72) † | | | 0.63 (0.34–1.18) | | |  |  |  |
| Academic achievement (above average vs. average or below) | | | | 1.00 (Reference) | | | 1.25 (0.94–1.66) | | | |  | 1.43 (1.03–1.99) † | | | 1.85 (1.24–2.76) ‡ | | | 2.13 (1.13–4.02) † | | |  |  |  |
| Reading distance (vs. < 20 cm) | | | |  | | |  | | | |  |  | | |  | | |  | | |  |  |  |
| 20–24 cm | | | | 1.00 (Reference) | | | 1.05 (0.73–1.51) | | | |  | 1.25 (0.79–1.96) | | | 0.95 (0.57–1.58) | | | 0.84 (0.38–1.86) | | |  |  |  |
| 25–29 cm | | | | 1.00 (Reference) | | | 0.85 (0.56–1.30) | | | |  | 1.55 (0.95–2.53) * | | | 0.84 (0.47–1.51) | | | 0.94 (0.39–2.24) | | |  |  |  |
| ≥ 30 cm | | | | 1.00 (Reference) | | | 1.01 (0.61–1.67) | | | |  | 1.05 (0.56–1.98) | | | 1.03 (0.51–2.08) | | | 0.54 (0.16–1.83) | | |  |  |  |
| Distance to television (vs. < 1.0 m) | | | |  | | |  | | | |  |  | | |  | | |  | | |  |  |  |
| 1.0–1.9 m | | | | 1.00 (Reference) | | | 1.28 (0.65–2.52) | | | |  | 1.02 (0.49–2.15) | | | 1.10 (0.44–2.76) | | | 0.54 (0.17–1.73) | | |  |  |  |
| 2.0–3.0 m | | | | 1.00 (Reference) | | | 1.46 (0.75–2.82) | | | |  | 1.01 (0.49–2.09) | | | 1.06 (0.43–2.60) | | | 1.08 (0.31–3.75) | | |  |  |  |
| > 3.0 m | | | | 1.00 (Reference) | | | 1.83 (0.90–3.72) * | | | |  | 1.49 (0.68–3.26) | | | 1.27 (0.48–3.37) | | | 1.49 (0.68–3.26) | | |  |  |  |
| Duration of near vision (per 1 h/day increment) | | | | 1.00 (Reference) | | | 0.93 (0.86–1.01) | | | |  | 0.96 (0.87–1.05) | | | 0.97 (0.86–1.08) | | | 0.94 (0.76–1.15) | | |  |  |  |
| Duration of outdoor activity (per 1 h/day increment) | | | | 1.00 (Reference) | | | 0.99 (0.80–1.22) | | | |  | 0.69 (0.52–0.92) † | | | 0.71 (0.52–0.99) † | | | 0.50 (0.25–0.98) † | | |  |  |  |
| Intake frequency of sugary drinks (≥ 1 vs. < 1 time/week) | | | | 1.00 (Reference) | | | 0.91 (0.63–1.30) | | | |  | 1.06 (0.71–1.60) | | | 1.51 (0.95–2.40) * | | | 1.28 (0.60–2.70) | | |  |  |  |
| Sleep quality scores (per 10 score increment) | | | | 1.00 (Reference) | | | 1.05 (0.90–1.22) | | | |  | 1.21 (1.02–1.43) † | | | 1.16 (0.92–1.46) | | | 1.14 (0.76–1.70) | | |  |  |  |
| Number of parents with myopia (per 1 increment) | | | | 1.00 (Reference) | | | 1.53 (1.26–1.85) ‡ | | | |  | 1.85 (1.47–2.32) ‡ | | | 2.36 (1.80–3.09) ‡ | | | 2.14 (1.41–3.25) ‡ | | |  |  |  |
| Mother's childbearing age (≥ 35 vs. < 35 years) | | | | 1.00 (Reference) | | | 0.93 (0.55–1.58) | | | |  | 0.63 (0.31–1.26) | | | 0.99 (0.48–2.05) | | | 1.20 (0.40–3.57) | | |  |  |  |
| Mode of delivery (caesarean vs. vaginal) | | | | 1.00 (Reference) | | | 0.97 (0.73–1.28) | | | |  | 0.80 (0.57–1.11) | | | 1.45 (0.98–2.17) * | | | 0.71 (0.37–1.34) | | |  |  |  |
| Duration of breastfeeding (< 6 vs. ≥ 6 months) | | | | 1.00 (Reference) | | | 1.03 (0.78–1.37) | | | |  | 0.92 (0.66–1.28) | | | 1.01 (0.68–1.50) | | | 2.00 (1.06–3.77) † | | |  |  |  |
| * *P*-value < 0.10.  † *P*-value < 0.05.  ‡ *P*-value < 0.01.  Abbreviations: RMB, renminbi (to convert to US dollars, multiply by 0.15). | | | | | | | | | | | | | | | | | | | | |  |  |  |


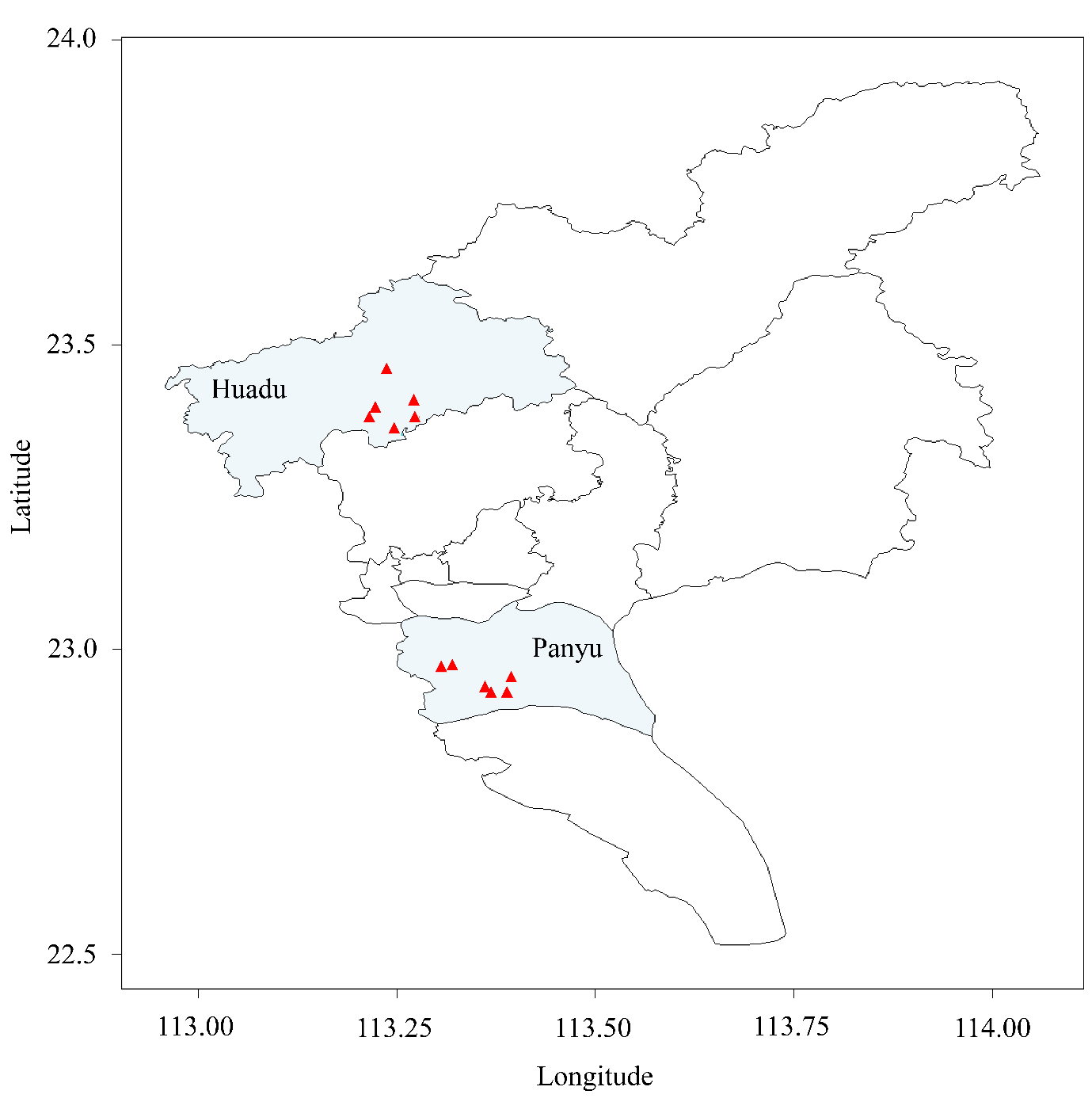


**Fig S1.** Map of schools' locations in Guangzhou, China


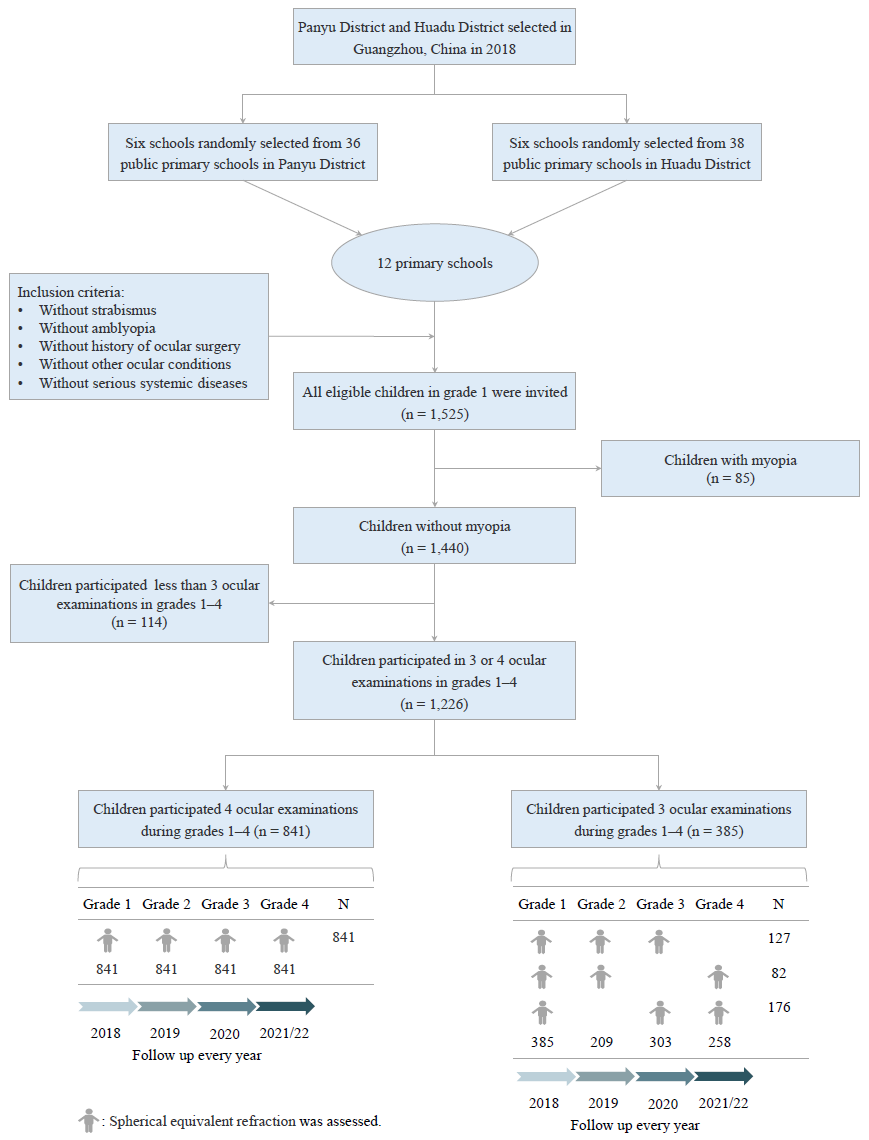


**Fig S2.** Flowchart of children included in analysis
